# An Edible H_2_O_2_ Biosensor for Gastrointestinal Metabolites and Peroxidase Enzyme Quantification

**DOI:** 10.1002/adhm.202503431

**Published:** 2026-04-02

**Authors:** Valerio Francesco Annese, Elena Feltri, Pietro Rossi, Valerio Galli, Cristiano Bortolotti, João Paulo Vita Damasceno, Ivan K. Ilic, Dario Natali, Alessandro Luzio, Adrica Kyndiah, Mario Caironi

**Affiliations:** ^1^ Center for Nano Science and Technology Istituto Italiano di Tecnologia Milan Italy; ^2^ Department of Physics Politecnico di Milano Milan Italy; ^3^ Dipartimento di Elettronica, Informazione e Bioingegneria Politecnico di Milano Milan Italy; ^4^ Department of Inorganic Chemistry Institute of Chemistry Universidade Estadual de Campinas Brazil

**Keywords:** edible electronics, edible biosensor, gastrointestinal tract sensing, H_2_O_2_ biosensor

## Abstract

Gastrointestinal (GI) fluids are a rich source of diagnostic biomarkers, yet access to these analytes remains limited to specialized clinical settings due to the rigidity, potential toxicity and environmental burden of conventional ingestible devices. Here, we present an edible biosensor for metabolite and enzyme activity quantification in gastric fluid, designed to be safely ingested and partially metabolized after use. The biosensor is validated for H_2_O_2_ quantification, a key reactive oxygen species associated with GI inflammations, via a controlled redox reaction involving caffeic acid and horseradish peroxidase. Following spectrophotometric and electrochemical validation, the redox system is integrated into an edible extended‐gate electrolyte‐gated field‐effect transistor that uses a toothpaste pigment as the semiconductor. The device is tested in vitro and detects H_2_O_2_ in the 0–3 mM range, with a limit of detection of ∼143.7 µM and sensitivity of 2.7 µC mM^−1^. As a proof‐of‐application, we demonstrate the use of the edible biosensor to detect metabolites (glucose and cholesterol) and biomarkers (gastric peroxide enzyme activity) by minimal modifications of the biorecognition elements, and we validate the sensing mechanism in simulated physiological environment. This work moves toward direct in vivo biosensing for the GI tract, which is safe and accessible at the point‐of‐care.

## Graphical abstract

We present an edible biosensor for gastric fluid analysis that integrates a caffeic acid–horseradish peroxidase redox system into an edible electrolyte‐gated transistor. The device enables rapid, low‐volume detection of H_2_O_2_ and, with minimal modification, metabolites and enzyme activity in simulated gastrointestinal conditions.

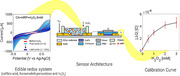

## 1 Introduction

Gastrointestinal (GI) fluids – including saliva, bile, gastric juice, pancreatic secretions, and intestinal fluids – represent an underexplored source of biomarkers, and swallowable devices provide a non‐invasive means to access them [1]. Since their introduction in 1957 [2], ingestible technologies have been employed to image and monitor GI tract physiology [3, 4, 5, 6, 7, 8, 9], yet despite a growing market projected to reach $1.96 billion by 2030 (CAGR 10.9%) [8], their development remains intrinsically limited by the materials used. [7] For implantable medical devices, the Food and Drug Administration (FDA) typically evaluates the entire device architecture rather than individual materials. As such, potentially toxic materials can be employed in ingestible systems with proper encapsulation. Most ingestible devices are rigid and non‐degradable, making adverse capsule retention a recognized clinical risk. [10] Retention rates vary depending on the device type and clinical indication, ranging from 0.3% [10] to 2.6% [7], and may necessitate surgical removal. Therefore, ingestible systems are typically deployed in a hospital environment by specialized staff rather than at the point‐of‐care (POC). Moreover, single‐use ingestibles also contribute to the accumulation of electronic waste (e‐waste) in wastewater and the environment.

Edible electronics, made from food and food‐additive materials, is an emerging research field that seeks to improve the safety of ingestible systems by using materials inherently safe for consumption [11]. These devices are designed to be degraded and/or digested, at least partially, after use, potentially eliminating device retention and enabling POC GI testing without additional e‐waste burden. The materials constituting an edible device are typically selected among those approved for human consumption by the European Food Safety Agency (EFSA) or the FDA, with conductive, semiconductive or insulating electronic properties [12, 13, 14]. Proof‐of‐concept edible electronic components (resistors [15], capacitors [16], transistors [17, 18, 19, 20], resonators [21], power sources [22, 23, 24, 25]), circuits [19], sensors (impedance [26, 27], strain [28, 29], pH, [30] temperature [31, 32], humidity [33, 34], and pressure [13, 35, 36]) and systems [37] are progressively emerging.

Edible devices offer the potential to safely access GI biomarkers in situ [38, 39, 40, 41], eliminating the need for sampling and subsequent analysis, which can introduce artefacts or degrade short‐lived biomarkers such as reactive oxygen species (ROS) [42]. ROS, including H_2_O_2_, have been linked to Inflammatory bowel diseases (IBDs), such as Crohn's disease and ulcerative colitis, affecting over 7 million patients worldwide [42]. GI enzymes associated with ROS, notably Myeloperoxidase (MPO) that infiltrates the gut during inflammation, are similarly linked to IBD [43, 44], systemic conditions [45], gastric ulcer [46], and colorectal cancer [47]. Other GI peroxidase enzymes include lactoperoxidase [48] and eosinophil peroxidase [49]. Furthermore, as with other point‐of‐care (POC) systems that leverage H_2_O_2_‐mediated pathways [50, 51, 52, 53, 54, 55], a wide range of metabolites can be indirectly quantified using H_2_O_2_ sensors functionalized with cascaded enzymatic reactions, including glucose [53], cholesterol [53], glutamate [54], and lactate [55]. Overall, H_2_O_2_ and its associated pathways are crucial for GI tract monitoring, as they reflect both metabolism and gastric enzyme activity.

In this work, we present an edible H_2_O_2_ biosensor for GI metabolites and peroxidase enzyme quantification. All materials used in the biosensor are safe for ingestion, being either food (or food‐derived) below the advised daily intake (ADI) or commonly ingested with no reported adverse effects. A thin film of ethyl cellulose (E462), a food additive approved by the EFSA, is used as the substrate. Gold (E175) and silver (E174) serve as electrodes. Copper phthalocyanine (CuPc), a cosmetic‐approved pigment commonly ingested without reported adverse effects [18], functions as the semiconductor. Chitosan, an edible polysaccharide from crustacean shells, is used as the primary electrolyte. Caffeic acid (CA) and Horseradish Peroxide (HRP) enzymes are used as biorecognition elements for H_2_O_2_ quantification. CA is a naturally occurring edible polyphenol found in fruits, vegetables, coffee, wine, and grains; peroxidase enzymes are naturally present in vegetables such as broccoli [56], potato [57], and turnip roots [58] and are involved in the process of food browning [57], For instance, the reported activity of peroxidase enzymes from a crude extract of broccoli is up to 53.2 U/mL [58, 59]. The biosensor features an extended‐gate electrolyte‐gated field effect transistor (ExG‐EGFET) architecture, where the sensing area (i.e. extended gate) is physically separated, although electrically connected, to the EGFET. Thus, when a signal is generated by the interaction between the sensor and the target analyte, it modulates the channel current, allowing transduction of the signal without exposing the transistor directly to the sample [60, 61, 62, 63]. The modular approach separates the biosensing area from the EGFET, allowing them to operate independently and avoiding potential compatibility issues. This configuration simplifies and protects the EGFET from chemical or biological degradation, and supports the use of diverse materials and geometries for the sensing electrode. The sensing mechanism is based on the oxidation of CA catalyzed by HRP in the presence of H_2_O_2_, which generates a signal transduced into a current output via the EGFET transconductance. The edible EGFET is essential in the absence of a miniaturized edible potentiostat. It buffers the signal, decouples sensing from transduction, and ensures a reliable, repeatable high‐impedance interface with the sensing area. After demonstrating the suitability of CA/HRP for H_2_O_2_ detection using traditional methods, we implemented and characterized the edible biosensor for in vitro H_2_O_2_ sensing. Like conventional systems, this platform is versatile, requiring only minor adjustments of the reagents to detect a range of metabolites and biomarkers. To illustrate this, we modified the secondary electrolyte for proof‐of‐concept sensing of glucose (with glucose oxidase), cholesterol (with cholesterol oxidase), and peroxidase (by reversing the reaction using H_2_O_2_ as a reagent). These results highlight the potential of future edible biosensors for in vivo GI tract monitoring, supporting personalized and digital health while reducing hospitalization needs.

## 2 Results and Discussion

### 2.1 Edible Redox System

**FIGURE 1:**
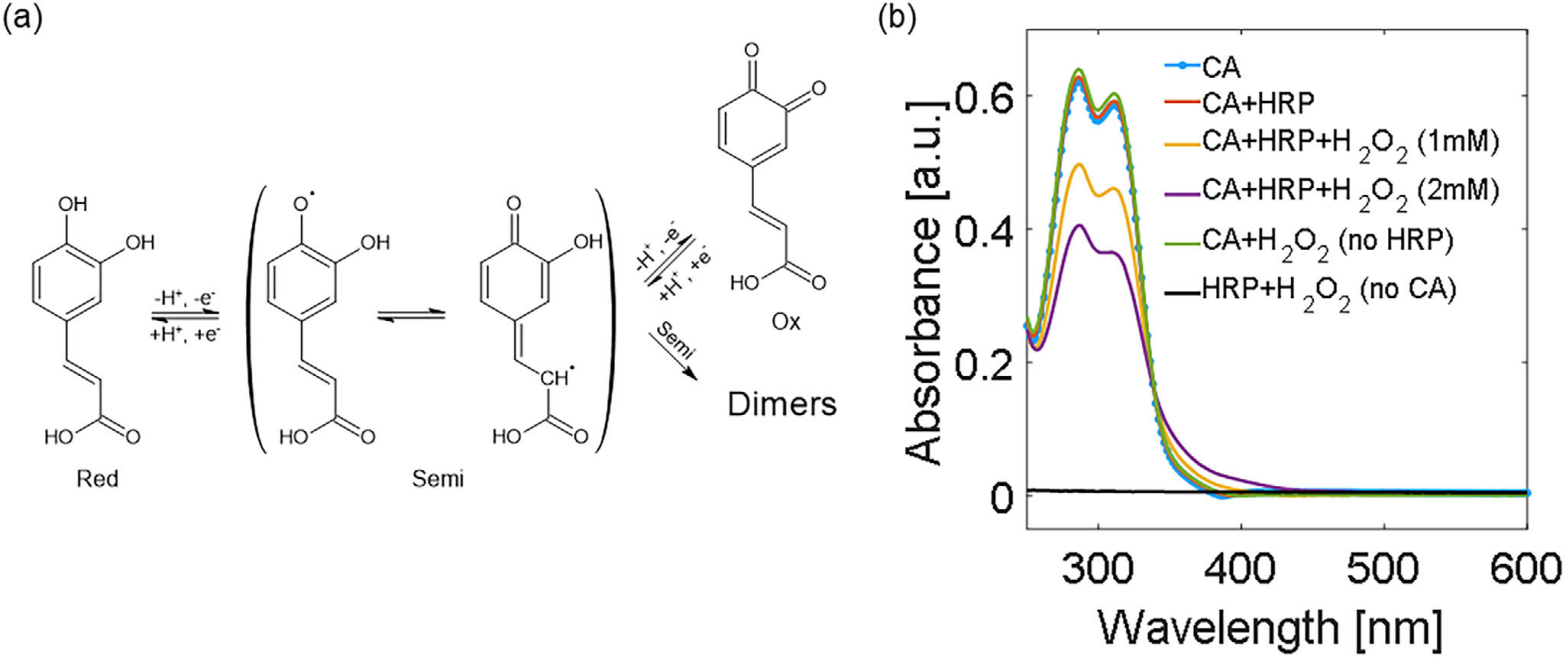
(a) Redox pathways for CA. After an initial formation of CA radicals, CA can be involved in either reversible oxidation or permanent dimerization [52]. (b) UV–vis spectra for diluted test samples in PBS (1:100).

Several chemical probes are available for H_2_O_2_ detection, with o‐dianisidine and phenol/antipyrine being widely used in laboratory settings [50]. However, these conventional redox systems are toxic upon ingestion, making them unsuitable for edible applications. The oxidation of CA in the presence of peroxidase and H_2_O_2_ was chosen to establish a stable, electrochemically active redox system compatible with edible sensing platforms. This choice is motivated by the syste0m biocompatibility, stability, and reliable electrochemical performance under physiological conditions. Upon oxidation, CA follows documented one‐ and two‐electron pathways leading to the formation of quinones, dimers, and polymeric structures (Figure 1a) [64]. Notably, the process is visually detectable due to the brownish coloration of the solution (Figure S1). HRP catalyzes this oxidation in the presence of H_2_O_2_ as per the following simplified reaction schematization [65, 66, 67, 68]:

(1) $$CA_{red}+H_{2}O_{2}\overset{HRP\;}{\to }\;CA_{ox}+H_{2}O$$

The redox reaction is composed of multiple steps. First, HRP reacts with H_2_O_2_ – which is reduced – to form an oxidized enzymatic intermediate. These highly reactive species then oxidizes CA by accepting electrons, generating CA radicals and oxidized products, while HRP returns to its neutral state [69].

We initially explored this edible redox system using UV–vis spectroscopy. We analyzed the UV–vis spectrum of CA (5 mM) in PBS in the presence of either H_2_O_2_ (1 mM) or HRP (10.9 U/mL) individually. Subsequently, we compared these spectra with the one resulting from the reaction of all three reagents with H_2_O_2_ (1 mM and 2 mM). Solutions were prepared and incubated for 1 h in dark conditions to avoid photo‐oxidation before being diluted in PBS (volume 1:100) to achieve a suitable optical density. The spectra, recorded in the 250–600 nm range, are shown in Figure 1b. The spectrum of CA displays two absorption peaks at 285 and 311 nm, assigned to electronic transitions consistent with the literature [70, 71]. The presence of an absorption tail above 340 nm indicates initial partial photo‐oxidation [70, 71]. When introducing only one of the two additional compounds in the solution (either H_2_O_2_ or HRP) the spectra remain very similar to the initial condition, with minimal differences to be ascribed to sample‐to‐sample variations. Only when all three chemical species are simultaneously present, an evident decrease in the intensity of the two main absorption peaks is observed. At the same time, the absorbance above 340 nm increases, indicating the enhanced formation of oxidation products (Figure S1). In the time analyzed within this study (1 h), the effect scales with H_2_O_2_ concentration and confirms the necessity of all three components for the oxidation reaction to occur. As a further control, the spectrum of a solution of H_2_O_2_ and HRP in PBS was acquired, obtaining in the investigated wavelength range a featureless response, therefore ruling out that any absorption peak could be linked to these two compounds or any product of their mutual interaction. Also, the spectra of CA in its oxidized form are in line with the literature [70, 71].

**FIGURE 2:**
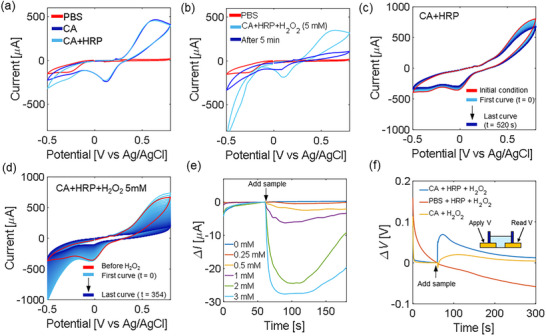
(a) CV of control samples: PBS (red), 5 mM CA in PBS (blue), and 5 mM CA in PBS with HRP (light blue). Scan rate 0.1 V/s. (b) H_2_O_2_ is added to a solution of 5 mM CA and HRP. CV immediately after adding H_2_O_2_ (light blue) is compared to the same curve after 5 min (blue), which resembles the PBS control (red). (c) Continuous CV with scan rate 0.5 V/s of a solution containing CA and HRP (no H_2_O_2_). (d) Continuous CV with scan rate 0.5 V/s monitored for more than 5 min. The last curve (blue) resembles the PBS curve, indicating that most of the CA has been consumed. (e) Two‐electrode (Ag/AgCl (RE/CE) and gold coil (WE)) chronoamperometry. The H_2_O_2_ sample is introduced after 60s. (f) Chronopotentiometry for two coplanar gold electrodes. *ΔV* is calculated with reference to the voltage value before inserting 1 mM H_2_O_2_ 60 s after starting the measurement. Inset: setup. A constant −0.5 V bias is applied at the control gate electrode, and the potential is measured at the second sensing electrode.

The investigation of the proposed edible redox system progressed to electrochemical analysis by cyclic voltammetry (CV) using a standard three‐electrode setup (platinum (counter electrode, CE), Ag/AgCl (reference electrode, RE), Au (work electrode, WE)). The PBS‐only scan exhibits a nearly featureless current, as expected, confirming the absence of significant redox activity (Figure 2a). The introduction of CA leads to a noticeable increase in current, with the appearance of oxidation and reduction peaks, indicating that CA can undergo electrochemical oxidation, likely forming its quinone derivative as described in the literature [72]. The further addition of HRP and H_2_O_2_ results instead in a rapid chemical oxidation, which consumes the redox‐active species faster than the electrochemical effect with CA only (Figure 2b). To explore the rate of the electrochemical response, continuous CV curves with a faster scan rate (0.5 V/s) were acquired. When only CA and HRP are analyzed, a slow and progressive decline of the reduction and oxidation peaks is observed (Figure 2c). This indicates a partial loss of electroactive species over time. However, when the experiment was repeated with the addition of H_2_O_2_ (Figure 2d), the peak currents decreased rapidly over time, with peak currents almost absent after only 5 min, indicating that H_2_O_2_ triggers a chemical redox process. This aligns with the expected peroxidase‐mediated oxidation of CA, where H_2_O_2_ serves as the electron acceptor. The observed loss of electroactive species is possibly due to the release of irreversible oxidation products [72].

The characterization progressed to chronoamperometry performed in a two‐electrode configuration using Ag/AgCl (RE/CE) and gold (WE), more similar to an ExG‐EGFET architecture (Figure 2e). A sharp current change occurs immediately after introducing H_2_O_2_ into the system close to the WE. As the analyte concentration increases, the current also increases, indicating higher current changes for higher concentrations. The 0 mM (blank) shows almost no change, confirming that the response is analyte dependent. After peaking, the current starts to recover. Increasing the concentration of the enzyme increased the initial speed of the reaction, while the applied voltage created differences in the dynamics of the reaction (Figure S2a,b). The analysis confirms catalytic oxidation in a two‐electrode configuration.

Finally, two‐electrode (gold‐gold) chronopotentiometry was adopted to mimic the extended gate configuration (Figure 2f). For this test, gold electrodes evaporated onto glass were used. A potential of −0.5 V is applied at the first electrode, and the potential is measured at the second electrode. After an initial wait time of 60 s, H_2_O_2_ is introduced into the system. As before, the condition producing a higher signal involves the use of CA and HRP. While in the absence of the oxidizing species, no clear signal is observed (orange), a smaller signal is also observed in the absence of the enzyme (yellow curve), possibly indicating that the reaction occurs even in the absence of the enzyme; however, the enzyme catalyzes it. Further tests of open‐circuit potential without bias also show a similar potentiometric response (Figure S2b,c). We speculate that the oxidation of CA in products/radical intermediates and the simultaneous reduction of H_2_O_2_ shift the electrochemical equilibrium at the sensing electrode. This temporary change in the redox environment alters the interfacial potential, resulting in a measurable variation in the open‐circuit voltage before recovery due to reagent consumption and diffusion of the oxidized species.

### 2.2 Edible ExG‐EGFET Platform

**FIGURE 3:**
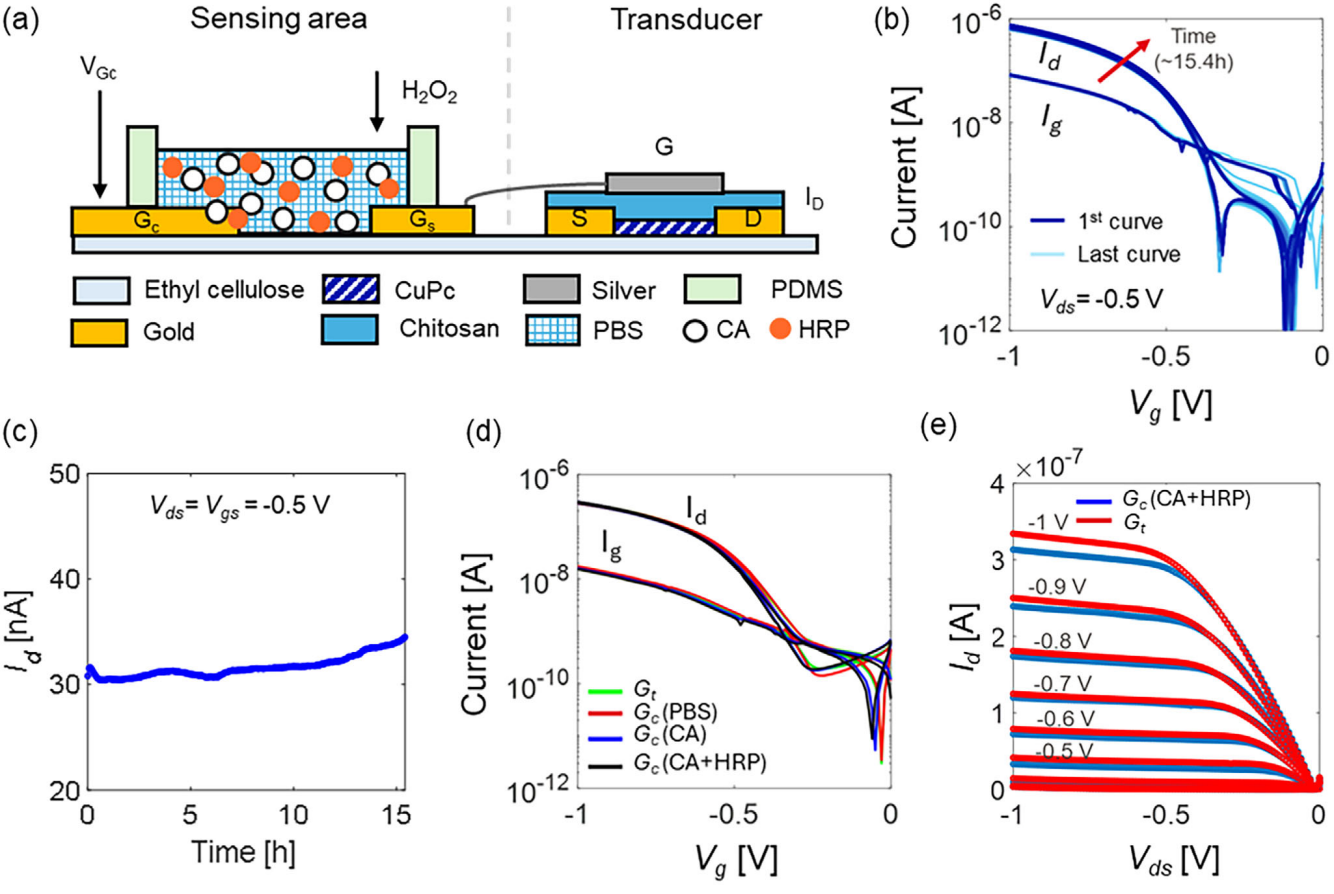
(a) Edible biosensor with extended‐gate architecture (not in scale). (b) Transfer curve (dark blue) from a representative device at *V_ds_* = −0.5 V from *G_t_*. To demonstrate the short‐time stability of the EGFET, the test is repeated for 15 h (3 min wait between two consecutive curves). The curves are overlapped, and only minimal deviations can be observed. (c) Stability of the drain current measured at a single bias point (*V_gs_* = *V_ds_* = −0.5 V) for more than 15 h extracted from the transfer curves. (d) Transfer curves for *V_ds_* = −0.5 V are compared when driving the EGFET from the *G_t_* (green) and the *G_c_* using PBS (red), 5 mM CA in PBS (blue) and 5 mM CA and HRP in PBS (blue) as the secondary electrolyte. (e) Output curves from *G_t_* (red) are compared to the curves obtained from *G_c_* (blue) using a solution of 5 mM CA and HRP in PBS as the secondary electrode.

The proposed edible redox system was adopted as the sensing mechanism in an ExG‐EGFET architecture. The edible biosensor is made of (i) the transducer (EGFET) and (ii) the sensing area (extended gate) connected in series (Figure 3a). Both were fabricated using only edible materials (see details in Supplementary Information). The edible EGFET was characterized in related works [18, 20]. The device is in a top‐gate bottom contacts configuration, with a thermally evaporated thin film of 20 nm of the p‐type toothpaste pigment copper phthalocyanine (CuPc) as the semiconductor, inkjet‐printed interdigitated gold contacts with channel width (W) and length (L) of 33 mm and 25 µm, respectively, inkjet‐printed silver as the top gate (*G_t_*), a drop‐casted chitosan‐based formulation as electrolyte, and ethyl cellulose as the substrate. The transfer characteristic curves of the device demonstrate the expected behavior of a p‐type transistor (Figure 3b, *V_ds_* = −0.5 V). The device shows a clear subthreshold region at low *V_gs_*, with exponential current rise and a high on‐state current around 10^−6^ A at *V_gs_* = −1 V. At *V_g_*_s_ ≈ 0 V, the current is in the picoampere range, reflecting low leakage and good off‐state performance. The transistor shows excellent stability in air, with absolute variations of the drain current measured at *V_gs_* = *V_ds_* = −0.5 V smaller than 4 nA over a 15‐h operation under bias (3 min wait time between each measurement, Figure 3b,c). The transistor also shows long‐term stability in air with a reported lifetime longer than 12 months [18].

The edible sensing area is composed of two gold electrodes printed onto ethyl cellulose immersed in a secondary liquid electrolyte where the redox system is in solution. The sensing gate (*G_s_* in Figure 3a) is connected to the top gate of the EGFET (*G_t_*) using standard probes/wiring. The dimensions of the extended gate are designed to maximize the voltage at *G_t_* when applying a bias from the control gate (*G_c_*), despite the voltage partition due to the series of capacitive components (electrical double layers) [61, 62, 63]. When operating the transistor at *V_ds_* = −0.5 V, the transfer curve is virtually identical when biasing the device from the *G_t_* or the *G_c_*. The device also operates similarly using different secondary electrolytes, namely PBS, CA in PBS and CA/HRP in PBS (Figure 3d). The output curves of the ExG‐EGFET biosensor only show minimal deviations when operating the device at higher voltages (e.g. *V_gs_* = −1 V, Figure 3e). Similar results are obtained if a non‐edible extended gate with the same geometry (evaporated gold onto Corning glass) is used in place of the edible one (Figure S3a).

### 2.3 H_2_O_2_ Sensing

**FIGURE 4:**
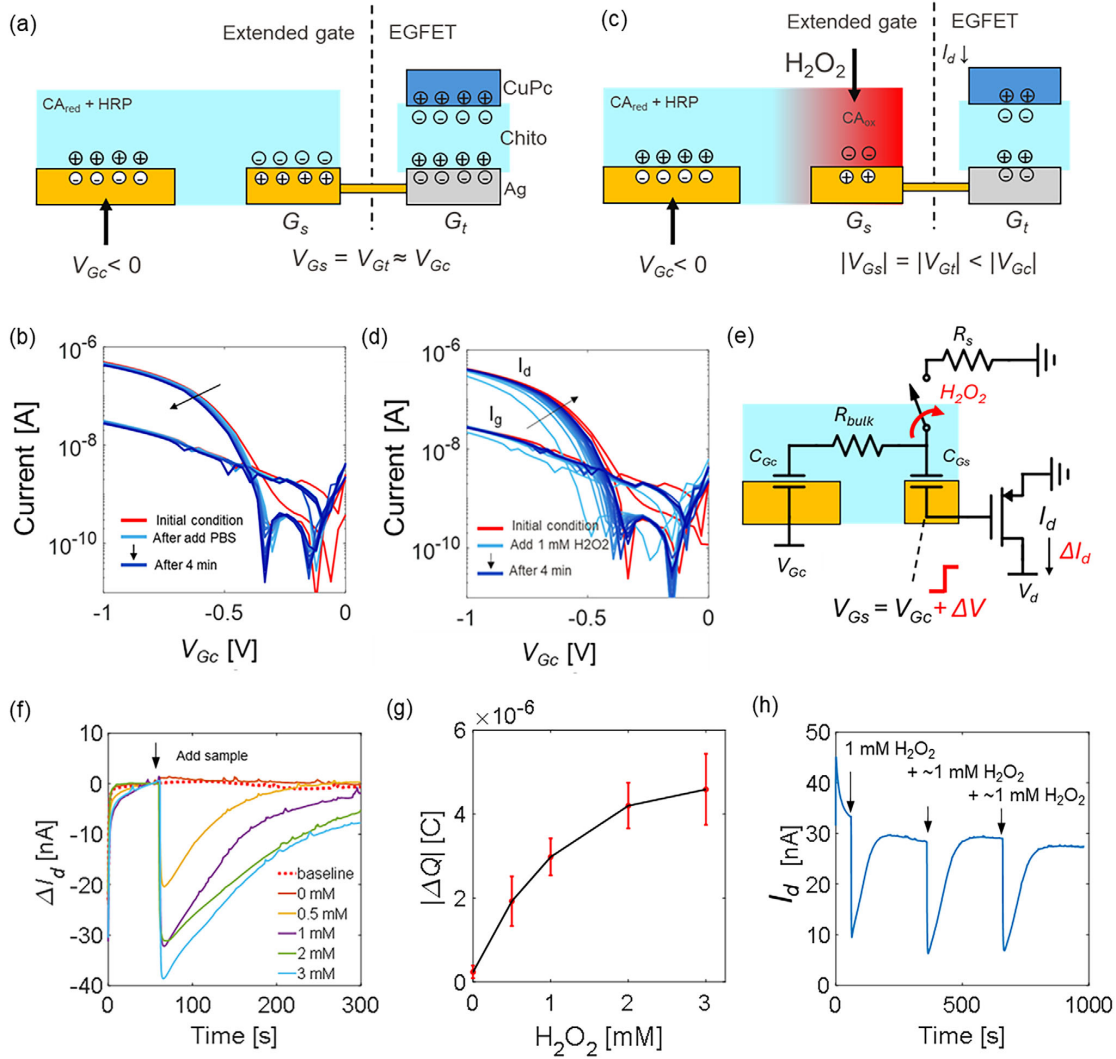
(a) Equilibrium condition of the edible biosensor. (b) No response upon insertion of a control sample (PBS) is observed_._ (c) Working mechanism of the edible biosensor upon introduction of H_2_O_2_. (d) The voltage decrease on the channel of the transistor creates a rigid shift of the characteristic when measured from the control gate. The curve gradually returns to the original condition in minutes. (e) Simplified equivalent electrical model of the biosensor. (f) Variations of the drain current at a specific bias point (*V_gs_* = *V_ds_* = −0.5V). The sample is introduced after a 60‐s equilibration time. (g) The average calibration curve and standard deviation of the biosensor are extracted by repeating the experiment over three different devices. (h) The biosensor provides a repeatable response when multiple samples of H_2_O_2_ are sequentially introduced into the system.

In equilibrium conditions and before introducing H_2_O_2_, the creation of electrical double layers and the proper dimensioning of the system ensure that the voltage at the EGOFET top gate (*V_Gt_*) matches the one applied at the control gate *G_c_*, namely *V_Gc_* (*V_Gs_ = V_Gt_ ≈ V_Gc_*, Figure 4a). In this state, the transfer curve of the transistor remains stable over time, even upon the addition of a control sample not containing H_2_O_2_ (Figure 4b).

When H_2_O_2_ is introduced near the sensing gate *G_s_*, chemical oxidation is triggered. The oxidized derivatives of CA, along with transient radical intermediates, release free charges that perturb the interfacial electrochemical equilibrium at *G_s_* temporarily reducing the source‐gate voltage of the EGFET, which in turn modulates *I_d_* (Figure 4c). To experimentally demonstrate the transduction, a transfer curve was repeatedly measured by sweeping *V*_Gc_ between 0 and −1 V (Figure 4d). A rigid shift of the transfer curve due to the variation of the effective voltage at *G_t_* with respect to the initial condition is observed after introducing H_2_O_2_ (from the red to the light blue curve). As the reaction progresses, a gradual recovery of the transfer is observed due to the exhaustion of reactants. A similar behavior is observed irrespective of the type of transistor connected to the sensing portion, for example, utilising carbon nanotubes instead of CuPc (Figure S4). The system is limited by diffusion, and the presence of a reversible electrochemical path (reduction of the oxidized species) is promoted by the availability of electrons at the *G_c_*. As such, when the reagents are consumed, a new equilibrium status is formed. The schematic in Figure 4e shows the simplified equivalent circuit of the biosensor. The extended gate is modelled as two interface capacitors (*C_Gc_* and *C_Gs_*) in series with the bulk electrolyte resistance (*R_bulk_*). Our sensor is designed with *C_Gt_* < *C_Gs_* < *C_Gc_* (Figure S8). When H_2_O_2_ is introduced, a switch closes, representing the onset of the redox reaction at the sensing gate, and generating the partial discharge of *C_Gs_* through *R_s_*. This produces a variation (*ΔV)* of the voltage applied to *G_t_*, causing a variation of the drain current (*ΔI_d_)*.

To simplify the readout of the sensor, we characterized the device at a single bias point. A constant *V_Gc_* = −0.5 V is applied to the *G_c_* while measuring the drain current *I_d_* over time with *V_ds_* = −0.5 V. In baseline conditions (no sample is introduced in the system), a small *I_d_* drift is observed over the 5‐min testing window (blue curve in Figure 4f). In the same condition, a liquid sample is introduced after a 1‐min wait time to reach a stable output. When the sample does not contain H_2_O_2_ (PBS only) only a small perturbation of the system is observed. Instead, when H_2_O_2_ is contained in the sample, a large and rapid current drop is observed. Increasing the concentration of H_2_O_2_ further, increases the current drop and elongates the recovery time. For large concentrations of H_2_O_2_ (3 mM), the biosensor starts to saturate.

To consider both the current drop and the recovery time and extract a calibration curve, the integration of the current was performed in a fixed 4‐min time window. This calculation corresponds to extracting the charge variation at the drain within 4 min from the introduction of H_2_O_2_. The experiment was repeated on three different devices (see Figure S5), and the average calibration curve was extracted (Figure 4g, Figure S6). The curve allows to estimate a limit of detection of 143.7 µM and sensitivity of 2.7 µC mM^−1^ in the range 0 – 1 mM obtained by linearization (*R^2^* > 0.98) (see Materials and Methods). The output of the sensor shows repeatability, with a recovery time < 3 min for 1 mM H_2_O_2_ (Figure 4h). It is worth noticing that the selected voltage level is compatible with edible batteries for future integration into a fully edible system [24].

## 3 Proof‐of‐Concept Applications

Generally, H_2_O_2_ sensors are highly advantageous because they can indirectly measure a large variety of biomarkers with minimal modification of the biorecognition elements. As such, three in‐vitro proof‐of‐concept applications exploiting the proposed edible biosensor are demonstrated, namely glucose, cholesterol, and simulated peroxide gastric enzyme sensing.

**FIGURE 5:**
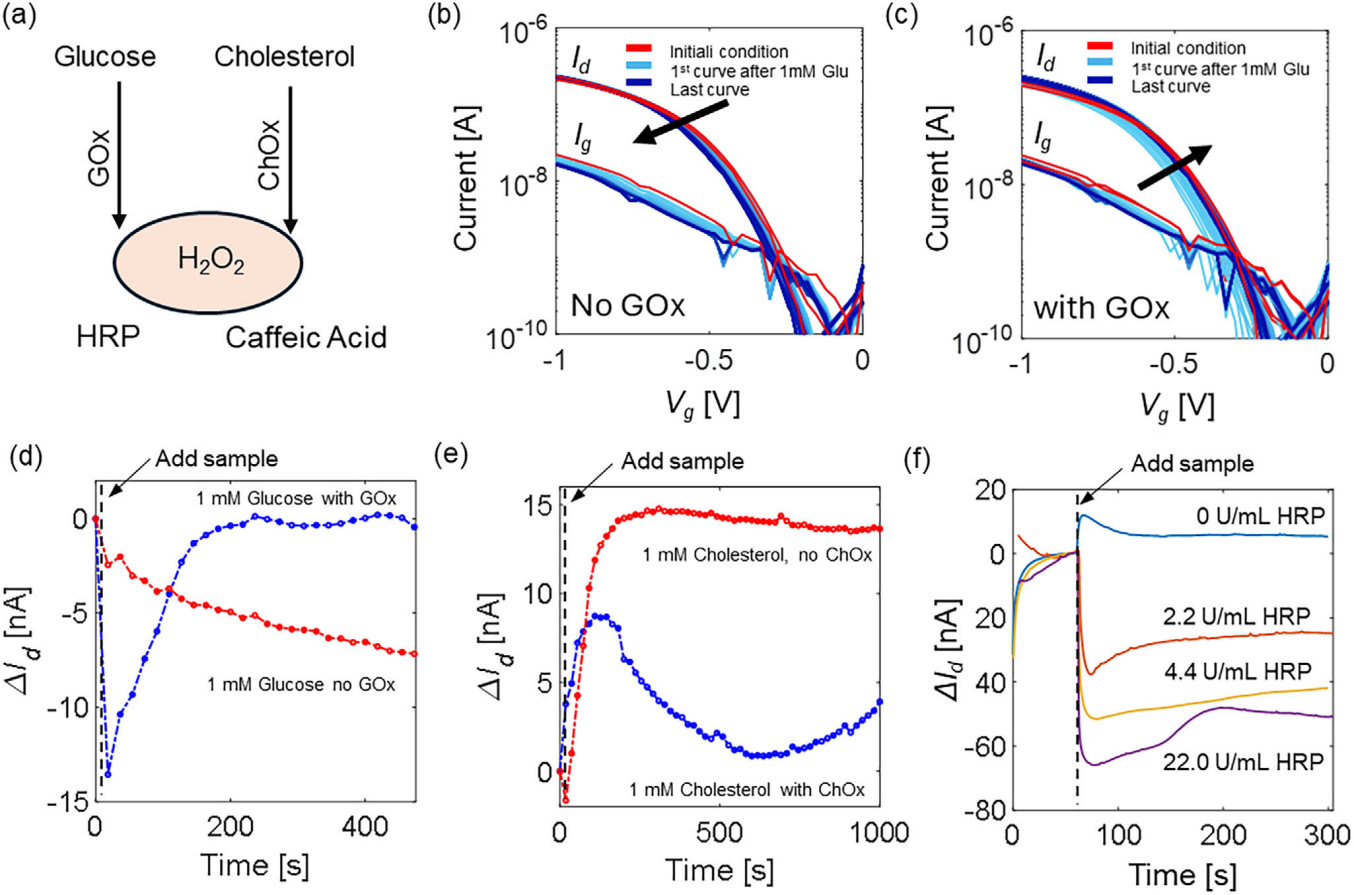
(a) Operation of the biosensor, where cascade enzymatic reaction produces H_2_O_2_ as a byproduct. (b) Transfer characteristics of the sensor exposed to 1 mM glucose in the absence of GOx. Total recording time ∼15 min. (c) Transfer characteristics of the sensor exposed to 1 mM glucose in the presence of GOx. Total recording time ∼15 min. (d) Δ*I_d_* of the sensor after glucose injection (1 mM), comparing conditions with and without GOx at *V_gs_* = *V_ds_* = −0.5 V. (e) Δ*I_d_* of the sensor after cholesterol injection (1 mM), comparing conditions with and without ChOx at *V_gs_* = −0.7 V and *V_ds_* = −0.5 V. (f) Δ*I_d_* vs HRP at *V_gs_* = −0.7 V and *V_ds_* = −0.5.

For glucose sensing, the secondary electrolyte was modified by the addition of glucose oxidase (GOx, E.C. 1.1.3.4). Thus, in the first reaction step, glucose reacts with GOx, producing H_2_O_2_. In turn, the H_2_O_2_ triggers the reaction with HRP and CA, producing the redox reaction, which constitutes the working mechanism of this biosensor (Figure 5a). GOx is commonly used in food processing [73], therefore the edibility of the platform remains valid. CV confirms the oxidation of CA when glucose and GOx are introduced into the system instead of H_2_O_2_ (Figure S7). Figure 5b shows the transfer characteristics of the biosensor upon exposure to 1 mM glucose without GOx enzyme. Minimal change in current after glucose addition indicates that H_2_O_2_ is not generated in the absence of GOx, confirming that the response of the sensor is enzyme‐specific. Differently, when the experiment is repeated with GOx, a substantial current shift is observed, reaffirming that the current modulation arises from H_2_O_2_ produced enzymatically (Figure 5c). The time‐dependent current response at a single bias point (*V_gs_* = *V_ds_* = −0.5 V) highlights the dynamic behavior of the sensor upon addition of glucose, closely resembling the response observed with H_2_O_2_ (Figure 5d).

A similar approach was used for cholesterol sensing by modifying the secondary electrolyte with the addition of cholesterol oxidase (ChOx, E.C. 1.1.3.6). ChOx is also used in the food industry [74]. The time trace depicts the current response upon exposure to 1 mM cholesterol with and without ChOx. A slow current shift is seen only with ChOx, demonstrating the capability of the sensor to detect cholesterol via the same H_2_O_2_‐mediated transduction route (Figure 5e). ChOx exhibits a slower reaction rate compared to GOx, resulting in a correspondingly slower and delayed current response.

Finally, the composition of the secondary electrolyte was modified to measure a HRP activity mimicking the MPO enzyme in the presence of CA and H_2_O_2_. In particular, 5 mM CA and 5 mM H_2_O_2_ were already preloaded in the sensing area, while HRP is the analyte introduced into the system. A high H_2_O_2_ concentration is introduced in the system to drive the sensor in saturation, also when a small amount of HRP is present. A current signal compatible with the ones observed during calibration is observed. The signal scales with the HRP activity, thus it can potentially be used for peroxide gastric enzyme quantification (Figure 5f).

## 4 Validation in Physiological Environment

To demonstrate the sensing mechanism, we employed buffered aqueous solutions to study the redox mechanisms involved, allowing us to decouple charge transfer effects from the core redox processes [74, 75]. However, the GI tract is a complex and heterogeneous environment. While its temperature is relatively constant (between 36.9°C and 37.4°C) [85], the GI tract is characterized by dynamic pH gradients, enzymatic activity, and the presence of various biomolecules that can potentially interfere with our biosensor. To provide an initial assessment of the biosensor in a physiologically relevant context, we conducted measurements in simulated GI conditions. We leveraged the modularity of the device architecture to independently test the transducer (edible EGFET) and sensing mechanism (edible redox system on extended gate fabricated with evaporated gold onto glass).

### 4.1 Materials Safety

We analyzed each material and related quantity used in the biosensor, as discussed in Table 1. By comparison with the reference intake established by international authorities, we conclude that all components of the transducer and extended gate are edible and used in amounts considered safe for ingestion.

**TABLE 1:** Estimated amount of each material per single device and corresponding ADI (or alternative reference intake value) as per EFSA (or other ingestion studies).

Material	Edibility	Functionality	Estimated Weight per device	ADI	Ref. and notes
Gold	Approved by EFSA as E175	Electrodes	47.3 µg	1.32 µg kg^−1^ day^−1^	[18, 77]
Silver	Approved by EFSA as E174	Top gate only	14 µg	12 µg kg^−1^ day^−1^	[18, 78]
Ethyl Cellulose	Approved by EFSA as E462	Substrate	30 mg	660–900 mg kg^−1^ day^−1^	[79]
Glycerol	Approved by EFSA as E422	Primary electrolyte	0.04 mg	No need for ADI	[80]
Chitosan	Approved as food material	Primary electrolyte	0.2 mg	3 g day^−1^	[81]
Copper Phthalocyanine	Approved in cosmetics such as toothpaste	Semiconductor	80 ng	N.A. An adult typically ingests < 1 mg day^−1^	[18]
Caffeic acid	Naturally present in food	Secondary electrolyte	0.41 mg	N.A. average 3.1 mg/100 mL in an espresso coffee	[82 – 84]
Horseradish peroxidase	Peroxidase enzymes are naturally present in food	Secondary electrolyte	21.9 U/mL	N.A. Extracted enzymes from broccoli have reported activity up to 53.2 U/mL	[59]

### 4.2 Temperature Effects

**FIGURE 6:**
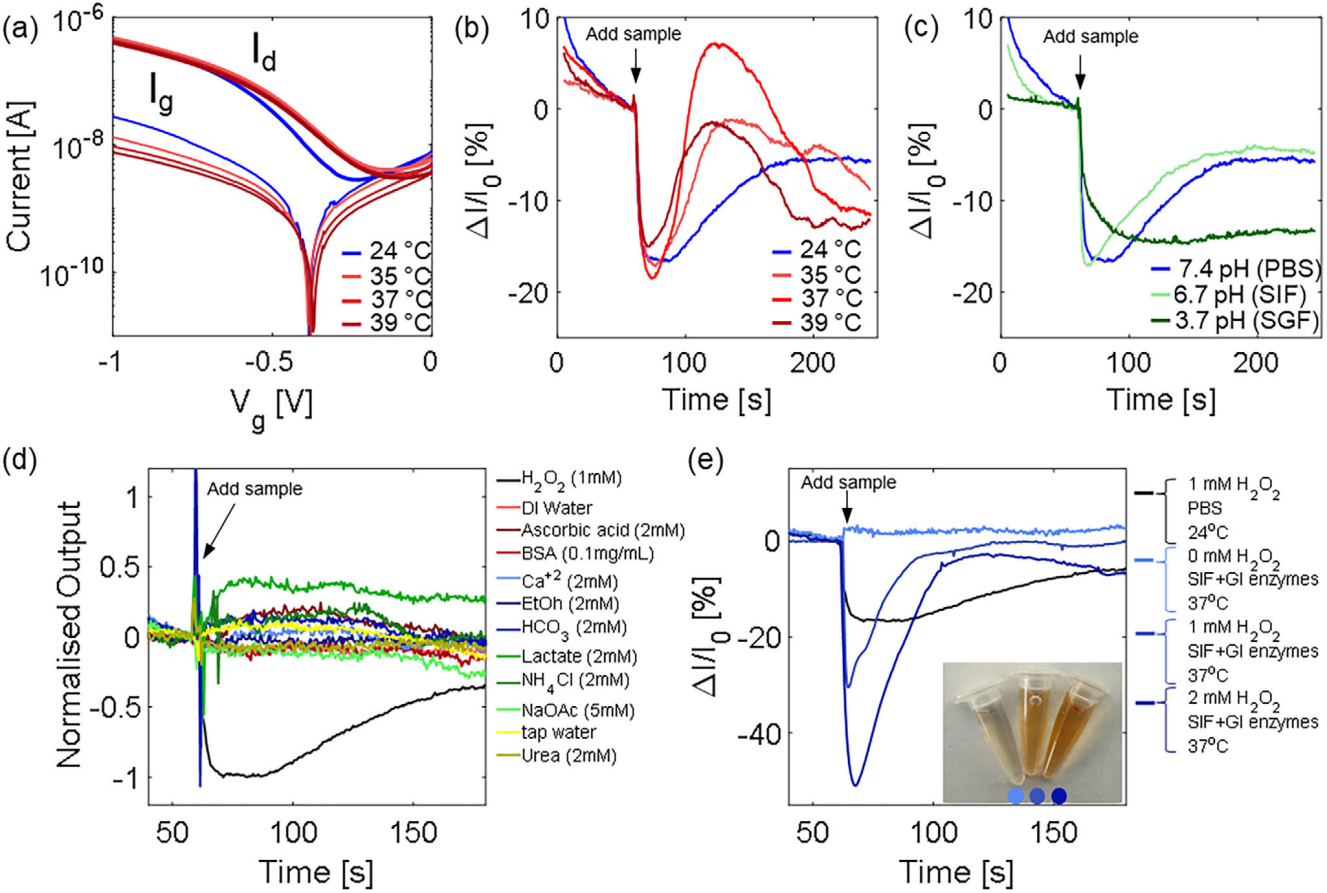
(a) Transfer of the device at physiological temperature (35°C, 37°C, 39°C) compared to room temperature (24°C). (b) Chronoamperometry of the extended gate to 1 mM H_2_O_2_ in PBS at physiological temperature (35°C, 37°C, 39°C) compared to room temperature (24°C). Each curve is an average over three experiments (single experiments are in Figure S9). (c) Chronoamperometry of the extended gate to 1 mM H_2_O_2_ at 24°C at different physiological pH levels, namely pH 7.4 (PBS), pH 6.7 (SIF) and pH 3.7 (SGF). (d) Chronoamperometry of the extended gate in PBS (pH 7.4) at 24°C to endogenous and non‐endogenous potential interfering substances. Signals are normalised to the average response of 1 mM H_2_O_2_, shown as a reference (black curve). The concentrations of the interfering substances are indicated in the legend and are higher than the reference in all cases except for BSA. (e) Chronoamperometry in physiologically relevant conditions, namely in SIF with additional GI enzymes (lipase, amylase, protease) at 37°C, compared to the response to 1 mM H_2_O_2_ in PBS at 24°C. Inset: the colour change observed under these conditions qualitatively indicates that the reaction continues to occur also in physiological environment.

Temperature affects both the transducer and the sensing mechanism. Figure 6a shows the EGFET transfer curves measured at physiological temperatures. Increasing the temperature leads to a small modulation of the drain current at the selected bias point (Figure S9a). Temperature also affects the redox reaction response (Figure 6b, Figure S9b–e). Comparing the amperometric response to 1 mM H_2_O_2_ in PBS at different temperatures, we observe a comparable initial response, with a slight increase in the maximum relative current variation from 15.5% at 24°C to 18.5% at 37°C (Figure 6b). However, different recovery dynamics are observed, with a tail current peak observed at 39°C and absent at 24°C (Figure S9b–e). Further tests are needed to understand the mechanisms of the temperature effects, but we speculate that higher temperatures may activate different redox pathways by altering kinetics, conformations, and transport. Nonetheless, these results confirm that physiological temperature influences both the electronic and electrochemical responses of the device, mainly affecting the recovery dynamics rather than sensitivity. Future strategies have to be adopted to compensate for temperature effects.

### 4.3 pH Effects

While the transistor can be encapsulated to avoid pH effects, the extended gate is exposed to the GI fluid. To assess the effect of pH, we assessed the amperometry response of the extended gate individually across different physiological pH levels (constant 24°C and 1 mM H_2_O_2_) in simulated intestinal fluid (SIF) and simulated gastric fluid (SGF). Experiments in PBS (pH 7.4) are instead comparable to the pH of saliva. In SIF (pH 6.7), the reaction rate increases during the initial phase, possibly due to enhanced HRP activity, while the recovery is virtually identical with respect to PBS. This is in line with reports of enhanced HRP activity in slightly acidic environments [86]. In SGF (pH 3.7), both the reaction and recovery rates are sensibly reduced, possibly due to the expected reduction of HRP activity at acidic pH levels [86]. Nonetheless, the current variation remains comparable to PBS and SIF. In both SIF and SGF, the signal is repeatable and scales with H_2_O_2_ concentration, confirming the responsiveness of the device (Figure S10). Overall, data show that pH affects the sensor dynamics; however, the sensor remains sensitive to H_2_O_2_ with comparable maximum current variations in all cases. Future investigations will focus on employing modified HRP [87] or alternative peroxidase enzymes (e.g. palm tree peroxidase [88]) to enhance stability against pH, temperature and time.

### 4.4 Interfering Agents

We tested the selectivity of the sensing mechanism against potentially interfering agents that are physiologically found in the GI tract, including endogenous (Ca^2+^, bicarbonate HCO_3_^−^, lactate, urea) and non‐endogenous (ascorbic acid, BSA, ethanol, ammonium chloride NH_4_Cl, sodium acetate NaOAc, tap water). Motivations and preparation details for the selected interfering agents are reported in Table S1. To this aim, we measured the amperometry response to each interfering agent of the extended gate in PBS at 24°C (Figure 6d). The concentration of the interfering agents was selected to be higher or equal to the reference (1 mM H_2_O_2_). Comparing the reaction of the interfering agents, in most cases, no observable deviation from the background was observed. The sensor output to lactate was the highest observed; however, it follows opposite dynamics and trend with respect to H_2_O_2_. Overall, data indicate that the sensing mechanism maintains selectivity for H_2_O_2_, with only minor responses to interfering substances, which is promising for the operation in complex matrices.

### 4.5 H_2_O_2_ Sensing in Simulated GI Environment

As a conclusive experiment, we verified the response of the sensor in advanced physiologic conditions. In particular, we modified the SIF with the addition of GI digestive enzymes, namely lipase, amylase and protease, and we performed the experiments at 37°C. Figure 6e demonstrates that, also in these physiological conditions, the relative current variations of the extended gate are responsive to the H_2_O_2_ concentrations and scale with its concentration. By comparison with the same experiment performed in PBS at 24°C, we observe a different dynamic response which modulates the shape of the pattern; nonetheless the signal remains detectable and scalable. The colour change of the solution confirms that the reaction proceeds under physiological conditions (inset Figure 6e).

### 4.6 Discussion

A colorimetric capsule for H_2_O_2_ detection based on a safe probe (Brilliant Blue FCF) has already been demonstrated [42]; however, it provides only qualitative information. In contrast, a quantitative capacitive edible H_2_O_2_ sensor was previously reported [40], but it functions as a passive component and operates within a higher detection range, and therefore lacks sensitivity at lower concentrations. Other POC [50, 54] and ingestible [89, 90, 91, 92] H_2_O_2_ sensing systems rely on rigid, non‐degradable components and, in some cases, require redox mediators which are not safe for ingestion, limiting their safety and applicability in POC context (see Table S2). The distinctive advantage of the present biosensor lies in its active sensing functionality, achieved entirely using materials approved as safe for ingestion, thereby combining biocompatibility, functionality, and safety within a fully edible platform.

Future studies will assess the safety of the integrated materials as a coherent system, including potential byproducts generated during degradation. The stability of the components under physiological pH and temperature for prolonged time will also be investigated. Further work will focus on preclinical validation to deepen understanding of the safety, functionality, and degradation behavior of the device under physiological conditions. In addition, the electrochemical mechanism and the specificity of the response, potentially limited by diffusion constraints at the electrode interface, will be further analyzed. Parallel efforts will aim at integrating the system into more complex ingestible and degradable platforms. A crucial step toward this goal will be its coupling with an edible power source, such as a miniaturized edible battery [23], to achieve fully ingestible and autonomous operation. Moreover, real‐time communication strategies based on human intra‐body communication [93, 94] will be explored to enable efficient, low‐power data transmission to external receivers, ensuring safe and unobtrusive monitoring outside the body.

## 5 Conclusion

H_2_O_2_ pathways in the GI tract are key indicators of both metabolic function and inflammatory activity, providing critical insight into gut health and disease. We present an edible biosensor based on an ExG‐EGFET architecture for the quantification of H_2_O_2_ and its application for metabolites (i.e. glucose and cholesterol) and peroxidase enzyme detection. The biosensor leverages an edible redox system composed of CA, H_2_O_2_, and peroxidase, enabling a robust and biocompatible electrochemical detection mechanism. The oxidation of CA in the presence of peroxidase and H_2_O_2_ was confirmed through UV–vis spectroscopy and electrochemical analysis, demonstrating its suitability as an edible redox system. The biosensor utilizes only safe‐to‐ingest and food‐approved materials, including ethyl cellulose as the substrate, gold/silver as the electrode material, and the toothpaste pigment copper phthalocyanine as the semiconductor. The ExG‐EGFET platform successfully transduced the biochemical reaction into an electrical signal, demonstrating its capability for H_2_O_2_ and peroxidase quantification in only 4 min and using a small sample volume of 0.5 mL. The device was validated in‐vitro in simulated physiological conditions. Such edible H_2_O_2_ biosensor provides a versatile platform for detecting a large variety of metabolites and biomarkers with minimal modifications of the secondary electrolyte. The device represents a first step toward in vivo edible biosensors for POC application.

## 6 Materials and Methods

### 6.1 Benchmark Analysis

To perform the UV–vis, samples are prepared first in Falcon vials and incubated at room temperature and in dark conditions for 1 h. Afterward, the samples were diluted to a 1:100 ratio in PBS and transferred to a quartz vial. Spectra were then measured using a double‐beam Perkin‐Elmer Lambda1050 spectrophotometer. All the electrochemical characterization was performed using a potentiostat (Multi‐PalmSens 4). For CV, a three‐electrode configuration was adopted, using a gold coil as the working electrode, a commercial Ag/AgCl (3 M KCl) as the reference electrode and a platinum coil as the counter electrode. The chronoamperometry was performed using two gold coils as electrodes. The open circuit potentiometry was instead performed using the same two coplanar gold electrodes geometry as the one used for the biosensor (Figure S8), but they were fabricated using thermal evaporation (Mbraun MB‐ProVap‐3) onto a Corning glass substrate. To fabricate these evaporated planar electrodes, Corning glass was cut into shape and used as a substrate. The first 5 nm chromium adhesion layer was evaporated through a custom aluminum mask. Chromium was then covered with a 30 nm layer of gold through the same mask. The size of the electrode is reported in Figure S8.

### 6.2 ExG‐EGFET

For the fabrication of fully edible transistors, a microscope glass slide was first coated with a thin layer of poly(dimethylsiloxane) (PDMS) to serve as an adhesion layer for the ethyl cellulose substrate, which was then carefully placed on top of the PDMS layer. The assembled stack underwent oxygen plasma treatment for 1 min to improve the wettability. Without additional processing, an interdigitated gold pattern (W = 33 mm, L = 25 µm) was directly inkjet‐printed with a Samba cartridge onto the ethyl cellulose film with a Fujifilm Dimatix DMP 2831, with the plate preheated to 60 °C. The pattern is then sintered on a hotplate at 120°C for 15 min. Next, a 20 nm layer of copper (II) phthalocyanine (CuPc) is deposited at a rate of 0.05 Å/s under a base pressure of 6 × 10^−^^7^ mmTorr in a thermal evaporation chamber (Moorfield Minilab L026) equipped with Low‐Temperature Sources (LTE). Following this, 3 µL of chitosan:glycerol solution was drop‐casted onto the interdigitated area, and the device underwent thermal treatment at 80°C for 30 min. A silver pattern was then inkjet‐printed on top to serve as the gate electrode and then sintered on a hotplate at 100°C for 15 min. For the preparation of the hydrated chitosan:glycerol electrolyte, chitosan (7.5 g/L) was dissolved in a 2% acetic acid aqueous solution and stirred overnight at room temperature. Glycerol is then added at 20% of the chitosan weight, and the solution is stirred for 20 min at room temperature before being stored at 4°C. The gold ink, DryCure Au‐J 1010B (10 cps, 10 wt%), was purchased from C‐INK Co., Ltd. Copper(II) phthalocyanine powder (99.9% purity), ethyl cellulose powder (48.0%–49.5% (w/w) ethoxyl basis), chitosan (low molecular weight product 448869), glycerol (≥99.5%), acetic acid (≥99.8%) and silver nanoparticle dispersion (30–35 wt% in triethylene glycol monoethyl ether) were purchased from Sigma‐Aldrich. PDMS (Sylgard 184) was prepared by mixing the precursor with its curing agent in a weight ratio of 10:1 and degassed in a vacuum chamber. The edible extended gate was printed using inkjet printing using the same ink and the same substrate as the EFGET. For testing purposes, another version of the extended gate was fabricated in gold on glass using thermal evaporation, as described above. To confine the fluid onto the extended gate, a cured PDMS pool was bonded onto the extended gate. The PDMS pool was cut to shape using a surgical blade (see Figure S8 for dimensions).

### 6.3 Redox system

For the redox reagents, CA (≥98.0%) and peroxidase from horseradish (∼150 U/mg) were purchased from Sigma‐Aldrich. 2.19 kU/mL aliquots of HRP in DI water were prepared and stored at −20°C. Single aliquots were thawed only once before use. H_2_O_2_ was purchased from Sigma‐Aldrich, stored in a refrigerator and freshly diluted to the desired concentration in DI water before use. A solution of 5 mM CA in PBS was prepared and stored in a closed amber vial in the refrigerator, and only for a short amount of time to avoid self‐oxidation.

### 6.4 Characterization

For electrical characterization, the transfer and output curves are measured using a B1500A Keysight Semiconductor Parameter Analyzer only in forward mode. The electrodes were contacted to the instrument using a probe station (Cascade Microtech) featuring precision micromanipulators and a microscope. To run the H_2_O_2_ assay, 450 µL of 5 mM CA and 5 µL of 2.19 kU/mL are first introduced onto the extended gate. The device is biased from the *G_c_* and the current is measured using the B1500A. After a wait time (60 s for the measurements at a single bias point), 45 µL of the sample solution containing variable quantities of H_2_O_2_ in PBS is introduced in proximity to the *G_s_* using a micropipette. To extract the calibration curve as charge variation (*ΔQ)*, current data in a fixed time‐window of 4 min is normalized (*ΔI = I – I_0_* with *I_0_* the current value just before the sample to be analyzed is introduced), rectified (| *ΔI |*) and integrated using the Matlab trapezoidal integration. The limit of detection (LOD) was quantified using the “International Union of Pure and Applied Chemistry” (IUPAC) definition [76]. The average (*µ_c_*) and standard deviation (*δ_c_*) of the *ΔQ* in the control experiment (only PBS) were 0.24 ± 0.15 µC. Consequently, the LOD (*µ_c_ + 3.3·δ_c_*) of the system is 0.735 µC. LOD expressed in Coulomb is then converted to µM by linearization of the calibration curve in the 0 – 1 mM range.

For metabolite sensing, glucose, GOx from Aspergillus niger (100 – 250 kU/mL), ChOx from Brevibacterium (≥50 units/mg) and water‐soluble cholesterol were purchased from Sigma‐Aldrich. Solutions were prepared in PBS in the following concentrations: ChOx 75 U/mL, GOx 1.5 kU/mL, glucose 40 mM, and cholesterol 40 mM. For the glucose assay, the assay formulation was (total volume 500 µL) 450 µL of 5 mM CA, 5 µL of HRP 2.19 kU/mL, 10 µL of GOx 1.5 kU/mL, and 35 µL of the test PBS solution (introduced after a wait‐time) containing glucose for a final concentration of 1 mM. For the cholesterol assay, the assay formulation was (total volume 500 µL) 450 µl of 5 mM CA, 5 µL of HRP 2.19 kU/mL, 12 µL of ChOx 75 U/mL, and 33 µL of the test PBS solution (introduced after a wait‐time) containing cholesterol for a final concentration of 1 mM. For peroxidase sensing, the assay formulation was (total volume 500 µL) 427.5 µL of 5 mM CA, 62.5 µL of 40 mM H_2_O_2_, and 10 µL of the PBS test solution (introduced after wait‐time) containing HRP.

### 6.5 Experiments in Simulated GI Conditions

SIF is a solution of 0.05 M KH_2_PO_4_ adjusted at pH 6.7 with a 0.2 M solution of NaOH without the use of any enzyme. CA was dissolved in the solution with a concentration of 5 mM. To modify SIF with GI enzymes, a water solution of GI enzymes was prepared by dissolving 15 kU lipase (15000 FIP), 6.12 kU amylase (amylase thera‐blend 8500 DU with estimated conversion 1 DU ≈ 0.72 U), 36 U protease (20000 HUT with estimated conversion 1 HUT ≈ 1.8 mU) in 100 mL DI water. Lipase, amylase and protease are purchased from Enzimedica in the form of food supplement (Lypo gold). The SIF solution with GI enzymes was prepared by mixing 420 µL of SIF with 30 µL of GI enzyme solution. The final concentration of the enzymes in the test solution is lipase 10 U/mL, amylase 4.08 U/mL and protease 0.024 U/mL. SGF is a solution of 0.03 M NaCl first adjusted at pH 1.4 with a 1 M solution of HCl without the use of any enzyme and subsequently diluted to pH 3.7 with DI water. CA was dissolved in the solution with a concentration of 5 mM. To bring the system to physiological temperature, we tested the devices using a precision temperature‐controlled stage (Linkam Scientific TMS94). The reference temperature reported in the figures is the stage temperature. The sample was left to thermally stabilize for over 5 min. To validate the sensing mechanism in simulated GI conditions, we used an edible transistor and multiple extended gates fabricated using evaporated Au on glass as described above. The sensing mechanism was studied using the B1500A Keysight Semiconductor Parameter Analyser. A constant voltage of −0.5 V was applied at the control gate, while current was recorded from the sensing gate.

## Author Contributions

V.F.A. conceived the study and designed the experiments with the support of A.L., A.K., and M.C. E.F. designed and fabricated the EGFETs. V.F.A. designed and fabricated the extended‐gate sensing electrodes. V.F.A. and P.R. performed UV–vis experiments. V.F.A., V.G., and C.B. performed the electrochemical characterization. J.P.V.D. and I.K.I. validated the interpretation of the electrochemical data. V.F.A., D.N., and M.C. developed and validated the electrical equivalent model. V.F.A. performed all electrical characterization and biosensing experiments.  A.K. supervised biosensing experiments with the support of A.L. M.C. supervised the project. V.F.A. drafted and revised the manuscript. All authors contributed to discussions and revisions.

## Conflicts of Interest

The authors declare no conflict of interest.

## Supporting information




**Supporting File**: adhm70806‐sup‐0001‐SuppMat.pdf.

## Data Availability

The data that support the findings of this study are available from the corresponding author upon reasonable request.
