# Supplementary material for: An Edible H2O2 Biosensor for Gastrointestinal Metabolites and Peroxidase Enzyme Quantification
Source: Adv Healthc Mater. 2026 Apr 2;15(21):e03431. doi: 10.1002/adhm.202503431 (PMC13241475; doi:10.1002/adhm.202503431)
Supplement: Supplementary file 1 — Supporting File: adhm70806‐sup‐0001‐SuppMat.pdf. [file ADHM-15-0-s001.pdf]

Supporting Information

An Edible H_2O_2 Biosensor for Gastrointestinal Metabolites and Peroxidase Enzyme Quantification

Valerio Francesco Annese, Elena Feltri, Pietro Rossi, Valerio Galli, Cristiano Bortolotti, João Paulo Vita Damasceno, Ivan K. Ilic, Dario Natali, Alessandro Luzio, Adrica Kyndiah, Mario Caironi**

V. F. Annese, E. Feltri, P. Rossi, V. Galli, C. Bortolotti, I. K. Ilic, D. Natali, A. Luzio, A. Kyndiah, M. Caironi
Center for Nano Science and Technology, Istituto Italiano di Tecnologia, Via Rubattino 81, Milan, 20134, Italy.
E-mail: valerio.annese@iit.it; mario.caironi@iit.it

V. Galli,
Department of Physics, Politecnico di Milano, Piazza Leonardo da Vinci, 32, Milan, 20133, Italy.

C. Bortolotti, D. Natali
Dipartimento di Elettronica, Informazione e Bioingegneria, Politecnico di Milano, Piazza Leonardo da Vinci, 20133, Milan, Italy.

J. P. V. Damasceno,
Department of Inorganic Chemistry, Institute of Chemistry, Universidade Estadual de Campinas, Brazil.

1. Edibility of Constituting Materials

The substrate of the biosensor is a thin film of ethyl cellulose. Ethyl cellulose is approved as a food additive by the European Food Safety Authority (EFSA) under the E number E462. Ethyl cellulose is commonly used in the food industry as a thickener, stabilizer, and coating agent. Source, drain, control gate and sensing gate are realized using gold. Gold is approved as a food additive by the EFSA as E 175. The top gate is instead realized using silver, also approved as food additive as E 174. Gold and silver are widely used in patisserie or gastronomy in the form of foils for aesthetic reasons. Copper phthalocyanine (CuPc), a synthetic pigment, is not considered edible and does not have an E number, although certain metal phthalocyanines are approved for use in cosmetics where part of the material can be ingested such as for instance in toothpaste. A recent study revealed that we ingest up to 1.0 mg of CuPc daily.[1] The primary electrolyte is a formulation containing chitosan, glycerol and water. Glycerol (E422) is a widely used food additive, serving as a humectant, sweetener, and solvent. Chitosan, a biopolymer derived from crustacean shells, is used in some dietary supplements and as a food preservative, though it is not assigned an E number. CA is a naturally occurring polyphenol found in coffee and various fruits, considered safe and often consumed in small dietary amounts. Enzymes such as horseradish peroxidase (HRP), glucose oxidase (GOx), and cholesterol oxidase (ChOx) are proteins used in biochemical assays; while not typically consumed, they are derived from natural sources and are non-toxic at the trace levels used in biosensors. Hydrogen peroxide (H_2O_2) can be used in food processing at very low concentrations and is rapidly decomposed into water and oxygen during enzymatic reactions. While CA is an edible and naturally occurring compound, the identity and potential physiological effects of its electrochemical oxidation products remain partially unresolved. However, it is important to note that similar reactions also occur naturally in food and within the human body.

2. Supplementary Figures and Tables

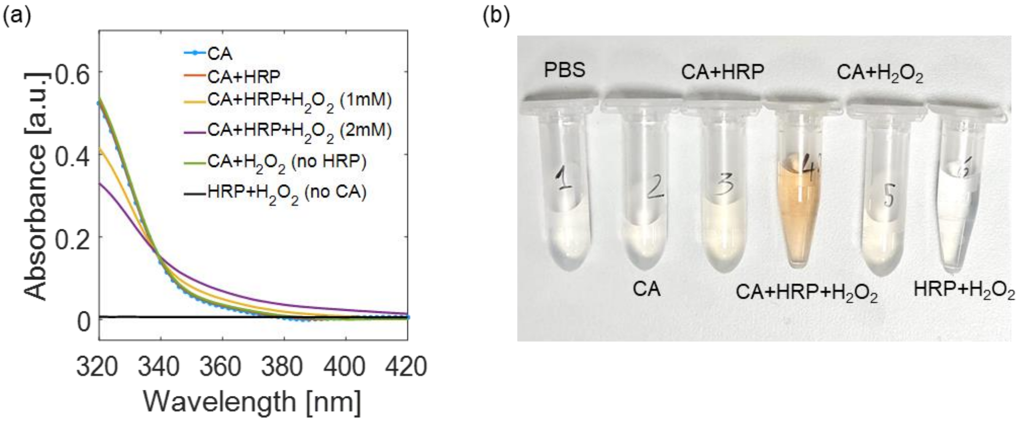

Figure S1. (a) UV-vis spectra for diluted test samples in PBS (1:100) in the range 320 – 420 nm. Only in the presence of both H_2O_2 and HRP, CA undergoes chemical oxidation (yellow and purple curves). This image complements the entire spectrum shown in the main paper to highlight the increase of light absorbance in the 340 – 400 nm range. (b) Demonstrative picture of the solutions before dilution 1:100 in PBS.

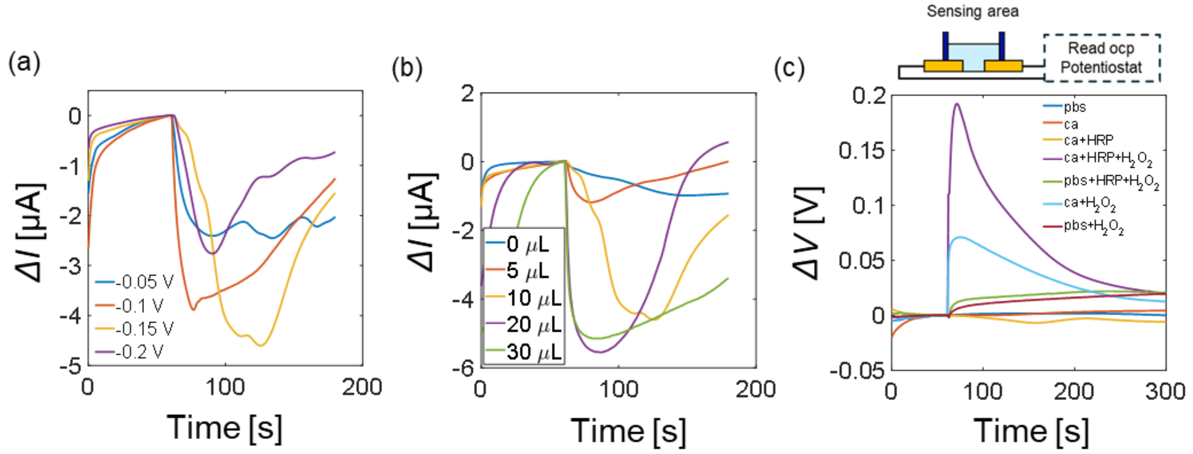

Figure S2. This image complements the two-electrode analysis reported in the main paper. ΔI and ΔV curves are obtained as $\Delta I = I - I_0$ and $\Delta V = V - V_0$ where I_0 and V_0 are the current and voltage measured at 60 seconds, just before the addition of the sample under test. (a) Chronoamperometry at different bias voltages. The reagent concentrations are fixed: CA 5 mM, HRP 1.1 U/mL, H_2O_2 500 µM. H_2O_2 is introduced after a 60-second wait time. (b) Chronoamperometry at different HRP volumes. Reagent concentrations: CA 5 mM, and H_2O_2 is 500 µM. The bias is also fixed for all the curves at -150 mV. HRP volumes/concentrations were: 0 µL / 0 U/mL; 5 µL / ~10.95 U/mL; 10 µL / ~21.9 U/mL; 20 µL / ~43.8 U/mL; 30 µL / ~65.7 U/mL. Total volume of the test was 1 mL. The concentration of the enzyme affects the speed of the reaction, and in particular, increasing the enzyme concentration decreases the time to the minimum of the curves. High concentration of the system saturates the system. (c) Open circuit potential with different combinations of the three reagents. The maximum output is observed when CA (ca) reacts with HRP and H_2O_2. In this experiment, H_2O_2 is 1 mM. Inset: Open circuit potential setup.

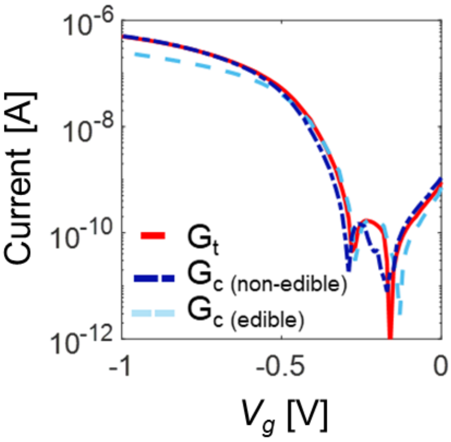

Figure S3. Comparison of the transfer curve of an edible transistor with V_{ds} = -0.5 V bias from the Gt and from the Gc. Biasing the transistor using an edible extended gate (Gc edible in figure) is comparable to biasing using a non-edible extended gate (Gc non-edible in figure). The edible extended gate is printed using gold on ethyl cellulose. The non-edible extended gate is gold evaporated onto Corning glass.

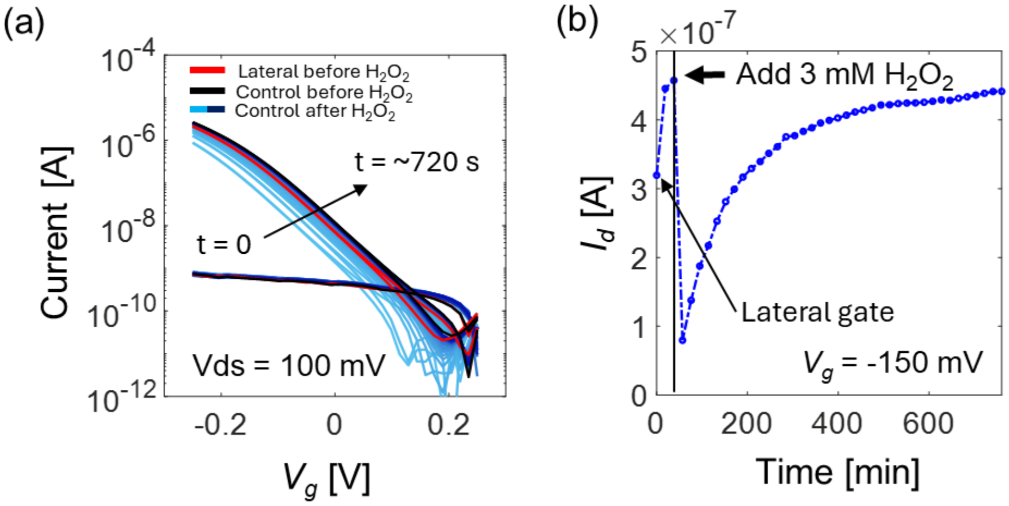

Figure S4. The transduction mechanism was repeated with a different transistor. In particular, we used a carbon nanotubes (CNTs) transistor. The CNTs transistor fabrication was carried out on glass substrates (Corning 1737F, Apex Glass LTD). To pattern the interdigitated source and drain contacts (W = 1800 um and L = 3 um), and the coplanar gate electrode (1.2 mm x 1.2 mm) a mask-less reverse lithography process (MicroChem GmbH AZ5214 photoresist together with an Heidelberg MLA100 mask-less aligner) was adopted. The gold metallic contacts (Cr 2 nm and Au 40 nm) were thermally evaporated (Mbraun MB-ProVap-3) and finally the lift-off process was completed by overnight immersion in TechniStrip® MicroD2. Before semiconductor deposition, the patterned substrates were cleaned sequentially in deionized water, acetone and 2-propanol (Sigma-Aldrich) submerged in an ultrasonic bath for 10 min each. Following, the substrates were exposed to a O_2 plasma (Femto Diener electronic) at 100 W for 5 min. The carbon nanotube-based ink was prepared, starting from monochiral single-wall carbon nanotubes sorted with poly[(9,9-dioctylfluorenyl-2,7-diyl)-alt-co-(6,6′-{2,2′-

bipyridine})] (PFO-BPy),[2] by ultrasound-assisted dispersion in 1,2-dichlorobenzene, according to the procedure reported in the literature.[3] The ink deposition was performed by ink-jet printing (Fujifilm Dimatix DMP-2850) on the channel area (9 layers, drop spacing = 20 µm). The printed semiconductor was annealed in air at 200 °C for 1 h, the wrapping polymer PFO-BPy was removed by soaking in n-butylacetate and consequently the samples were dried under a nitrogen flux. The solid-state electrolyte was prepared in air by mixing the co-polymer poly(vinylidene fluoride-co-hexafluoropropylene) (PVDF-HFP, Merck) and the ionic liquid 1-Ethyl-3-methylimidazolium bis(trifluoromethylsulfonyl)imide (EMIM:TFSI, Merck) in acetone (17.6 wt % ionic liquid, 4.4 wt % polymer, and 78 wt % solvent). The solution was stirred at 40°C for 1 h and then directly drop-casted on the gate-channel of the devices.

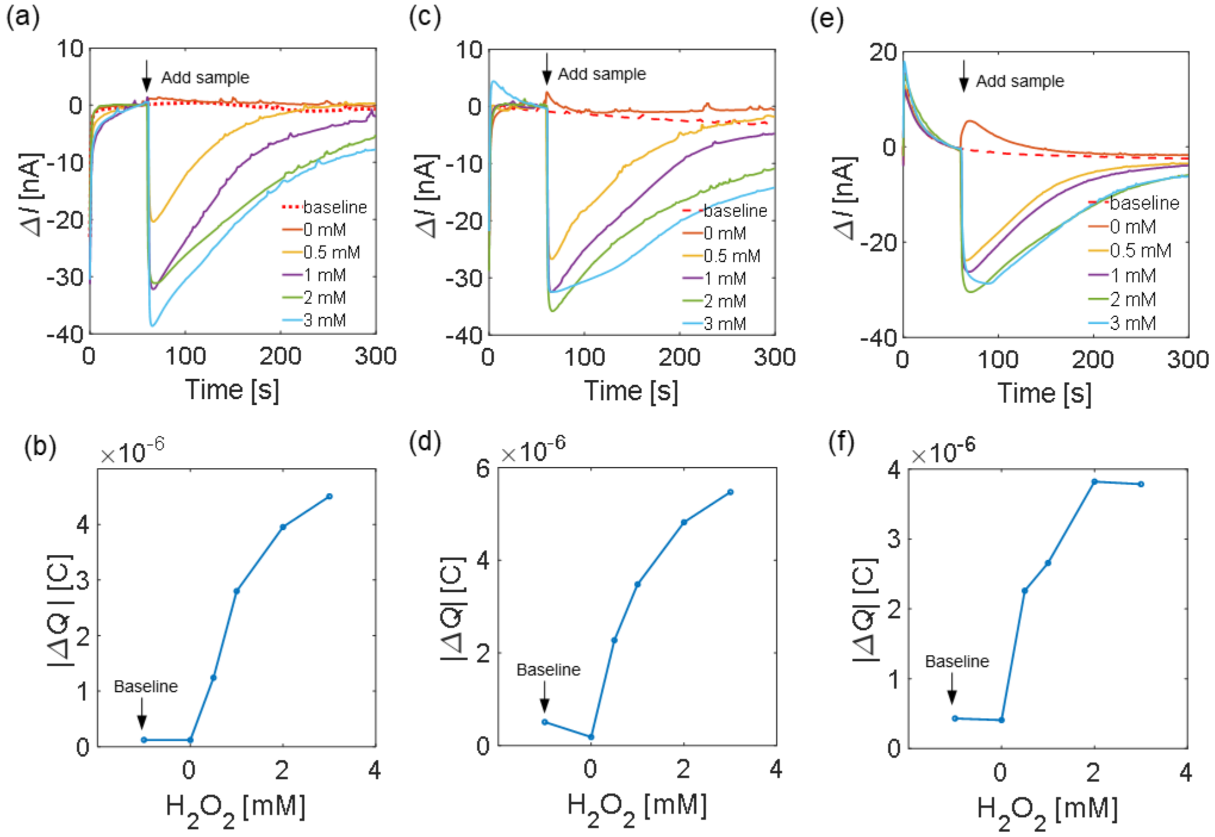

Figure S5. Dataset for three different biosensors. (a)-(c)-(e) Raw data from the biosensor normalized to the current value recorded at 60 seconds i.e. when the sample is added into the system. (b)-(d)-(f) Extraction of the charge variation for each of the replicates.

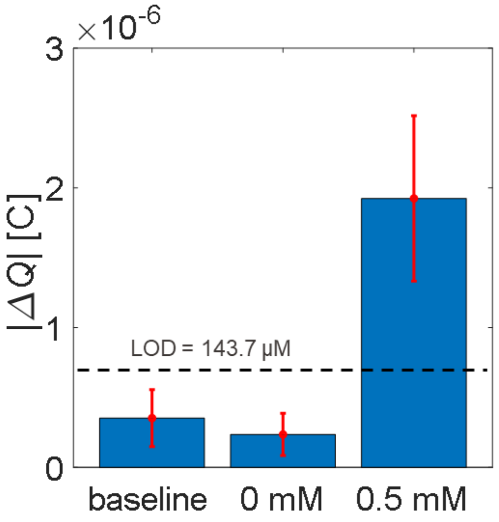

Figure S6. Representation of the absolute charge variations and limit of detection compared to baseline measurements (drift), the blank condition (0 mM H_2O_2), and signal from 500 µM H_2O_2.

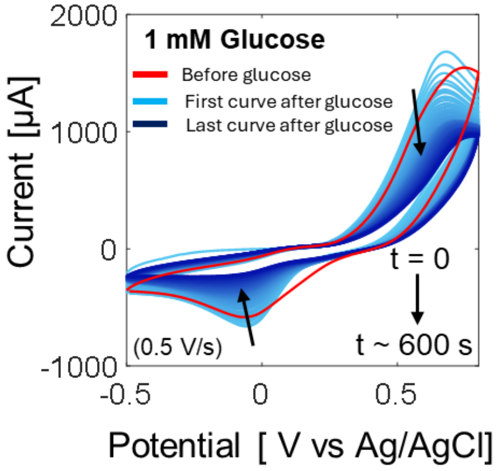

Figure S7. Cyclic voltammograms (CVs) during the enzymatic reaction between glucose and glucose oxidase (GOx), producing hydrogen peroxide (H_2O_2), which subsequently oxidizes CA in the presence of horseradish peroxidase (HRP). The red curve represents the CV before glucose addition. The light blue curve shows the first scan immediately after glucose introduction (t = 0), while the dark blue curve corresponds to the final scan after ~600 s (scan rate: 0.5 V/s). Over time, the progressive decrease of the oxidation current peaks of CA resulting from the enzymatic cascade reaction is observed.

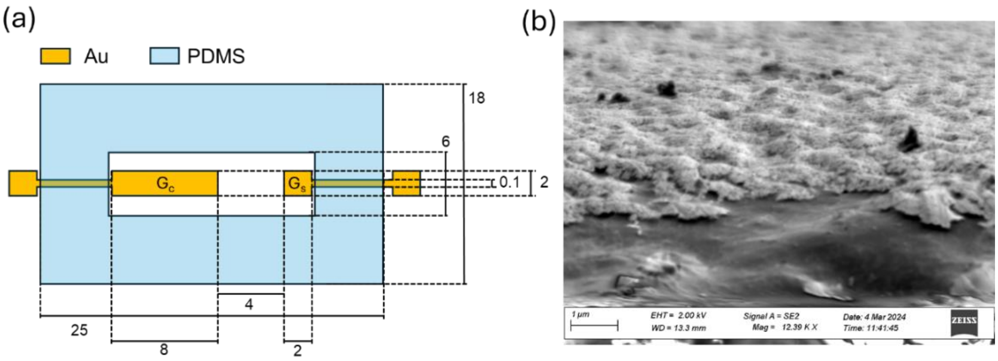

Figure S8. Dimensions of the gold extended gate. The edible extended gate (printed gold onto ethyl cellulose) and the evaporated one (evaporated gold on Corning glass) have the same design. The dimensions are expressed in millimeters. (b) Scanning Electron Microscopy (SEM) section of gold printed on the ethyl cellulose substrate.

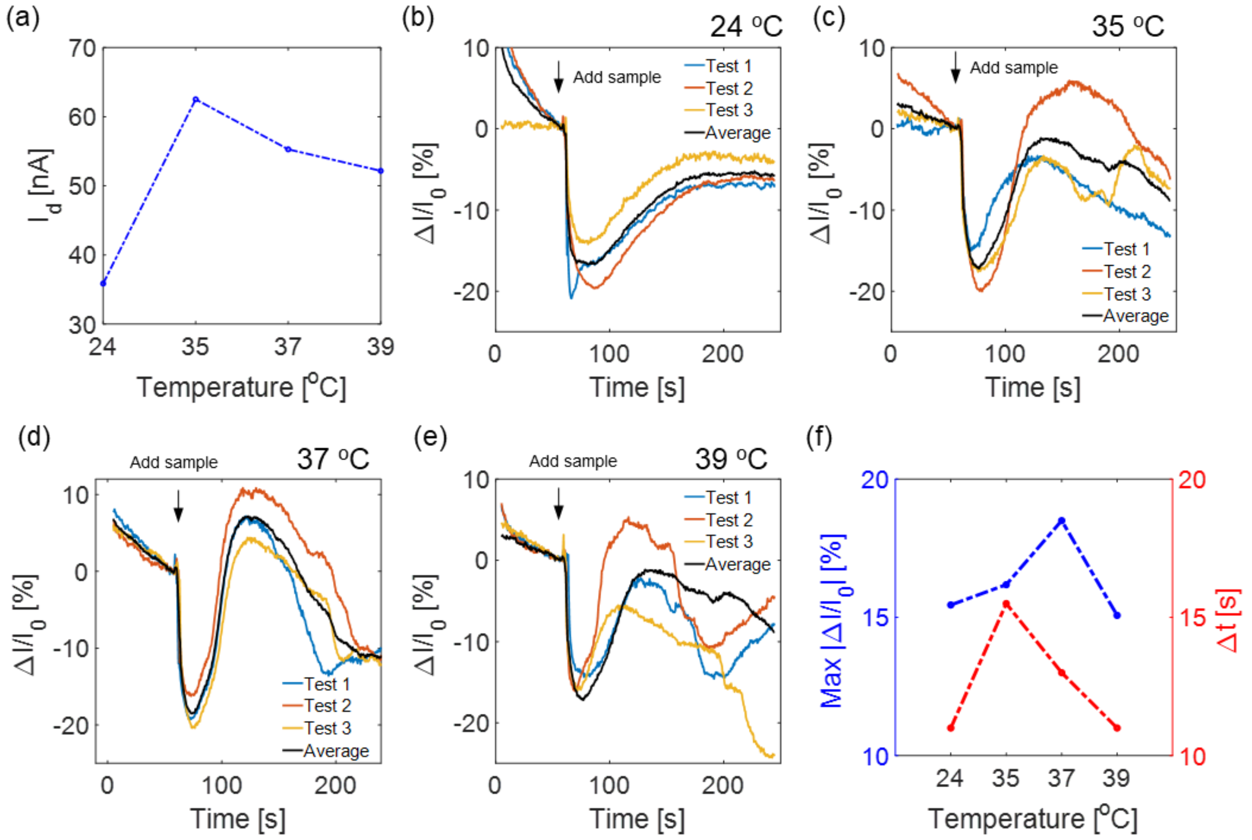

Figure S9. Temperature effects. (a) The drain current of the transistor at the working bias point temporarily increases when the temperature is increased from 24°C to 35°C. When the temperature is further increased to 37°C and 39°C, the drain current is reduced with respect to 35°C, probably due to degradation of the primary electrolyte. (b) Amperometry response of the extended gate architecture to 1 mM H_2O_2 without the transducer for three different trials and the average in PBS at 24°C, (c) 35°C, (d) 37°C and (e) 39°C. (f) Comparison of the maximum relative current variation (blue) and time required to reach the maximum (red) for the tested temperature levels.

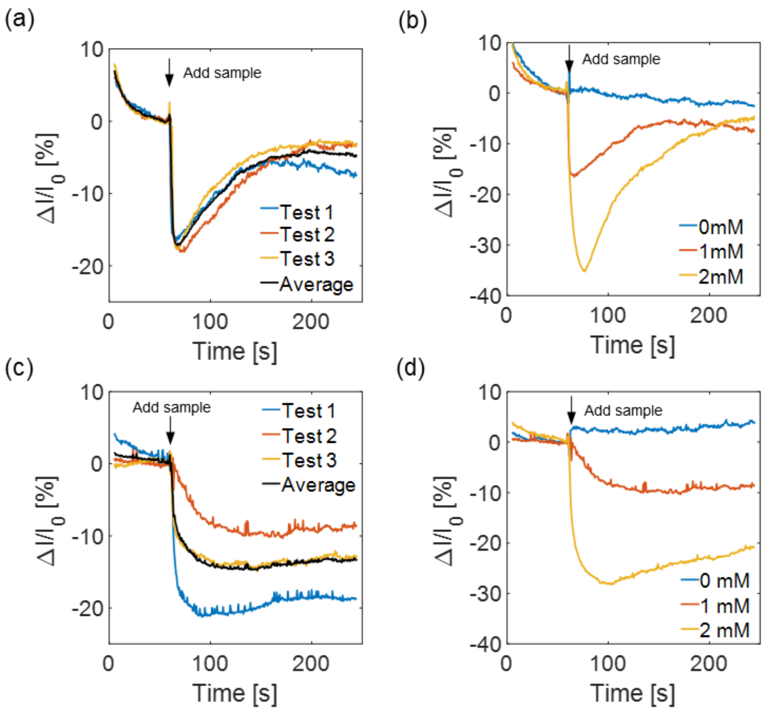

Figure S10. pH Effects. (a) Amperometry response of the extended gate architecture to 1 mM H_2O_2 without the transducer for three different trials and their average at 24°C in SIF (pH 6.7). (b) Amperometry response in SIF at 24°C to 0 mM, 1mM and 2mM of H_2O_2. (c) Amperometry response of the extended gate architecture to 1 mM H_2O_2 without the transducer for three different trials and their average at 24°C in SGF (pH 3.7). (d) Amperometry response in SGF at 24°C to 0 mM, 1mM and 2mM of H_2O_2.

Table S1. Potentially interfering agents. The motivation and preparation process of each selected agent to assess the selectivity of the platform is reviewed in the table. All the chemicals were purchased from Sigma.

Interfering agent	Source	Potential Interference	Preparation of the Stock Solution
Ascorbic acid (vitamin C)	Non-endogenous in humans, but is endogenous in several animals. Humans assume ascorbic acid from dietary intake in relatively large amounts. The concentration of ascorbic acid in gastric juice is < 0.5 mM.[5]	It can i) donate electrons and reduce oxidized caffeic acid back to its original form; ii) react directly with H_2O_2, reducing substrate availability for HRP.	Ascorbic acid solution in DI 40 mM.
Calcium ions (Ca^{2+})	Endogenous in humans, though dietary intake may be needed to maintain adequate levels.	It can i) reduce enzyme stability; ii) bind to carboxylate groups of phenolic acids like caffeic acid, thus reducing the availability of free caffeic acid.	10 mM $CaSO_4$ solution in 10 mM KCl

Bicarbonate (HCO_3^-)	Endogenous in humans, but can also be obtained from dietary sources or administered medically.	It can i) shift the pH and modulate HRP activity; ii) react with H_2O_2 to form carbonate radicals ($CO_3^{\bullet-}$) under some conditions. And reduce the effective concentration of H_2O_2	100 mM $NaHCO_3$ solution in 10 mM KCl
Lactate	Endogenous metabolite produced during anaerobic glycolysis; also present in foods and beverages. Lactate levels in the GI tract are below 5 mM.[6]	It can (i) act as a mild reducing agent, potentially competing with caffeic acid for oxidation; (ii) may alter local pH or ionic strength, affecting HRP activity.	Lactate solution 100 mM in DI water.
Urea	Endogenous metabolite of protein catabolism; excreted in urine and present in biological fluids. Gastric urea concentration can be up to 6 mM.[4]	(i) Can partially denature HRP at high concentrations, altering enzyme conformation; (ii) may interfere with hydrogen bonding and protein–substrate interactions.	Urea solution 100 mM in DI water.
Bovine Serum Albumin (BSA)	Non-endogenous protein commonly used as a model biomolecule or stabilizer in biological assays.	(i) Can adsorb to enzyme or electrode surfaces, blocking active sites; (ii) may non-specifically bind caffeic acid, decreasing free substrate availability.	1 mg/mL BSA solution in 10 mM KCl.
Ethanol	Non-endogenous compound commonly ingested in beverages.	i) Can alter HRP tertiary structure and reduce enzymatic activity; (ii) changes solvent polarity, potentially affecting caffeic acid solubility and reaction kinetics.	100 mM ethanol solution in DI water.
Ammonium chloride (NH_4Cl)	Endogenous in trace amounts via nitrogen metabolism; also Non-endogenous from diet or environment.<br>Gastric ammonia levels have been reported up to 4 mM.[4]	(i) Alters ionic strength and local pH; (ii) ammonium ions may compete with H^+ in the HRP catalytic cycle, modulating activity.	100 mM NH_4Cl solution in DI water.
Sodium acetate (NaOAc)	Common metabolic intermediate and food additive; also used as a buffering agent.	(i) Alters pH and buffer capacity, affecting HRP activity; (ii) acetate ions may weakly complex with phenolic substrates.	Sodium acetate buffer solution 5 mM purchased from Sigma.
tap water	Non-endogenous source containing variable ions (Ca^{2+}, Mg^{2+}, Cl^-, HCO_3^-, etc.) and impurities.	(i) Ionic impurities may affect HRP activity and substrate oxidation rate; (ii) may introduce trace metals that catalyze side reactions with H_2O_2.	Tap water sourced from our facility in Milan, Italy.

Table S2. Comparative table highlighting the differences between this platform and existing in vitro H_2O_2 biosensors.

Analyte	Sensing Mechanism	Application	Safety	Metrics	Ref
L-type amino acids, glutamate, choline, sarcosine (via H_2O_2 pathways)	Colorimetric	Point-of-care	Materials are not safe to ingest. Non degradable	LOD = 1.4 μM (for glutamate)	[7]
H_2O_2	Amperometric	Point-of-care	Degradable	LOD = 6.31 μM	[8]
H2O2	Electrochemical	n.d.	Materials are not safe to ingest	S = 116 μA μM^{-1} cm^{-2}	[9]
H_2O_2	Capacitive	GI Tract	Fully edible	n.d.	[10]
H_2O_2	Colorimetric	GI Tract	Degradable	Only qualitative measurements	[12]
H_2O_2	Luminescence	GI Tract	Non degradable	n.d.	[13]
MPO	Chemiluminescence	GI Tract	Partially degradable	S = 6.14 μJ/U.ml^{-1}	[14]
H_2O_2	Electrochemical	GI Tract	Non degradable	S ~ 0.15 nA μM^{-1}	[15]
H_2O_2	Electrochemical	GI Tract	Partially degradable	LOD = 242 nM	[16]
H_2O_2	Electrochemical	GI tract	Fully edible	LOD = 143.7 μM	This Work
LOD = limit of detection; S = sensitivity.					

References of the Supplementary Information

[1] Feltri, Elena, Pierluigi Mondelli, Bojan Petrović, Fabrizio Mario Ferrarese, Alina Sharova, Goran Stojanović, Alessandro Luzio, and Mario Caironi. "A fully edible transistor based on a toothpaste pigment." *Advanced Science* 11, no. 41 (2024): 2404658.

[2] Graf, Arko, Yuriy Zakharko, Stefan P. Schießl, Claudia Backes, Moritz Pfohl, Benjamin S. Flavel, and Jana Zaumseil. "Large scale, selective dispersion of long single-walled carbon nanotubes with high photoluminescence quantum yield by shear force mixing." *Carbon* 105 (2016): 593-599.

[3] Molazemhosseini, Alireza, Fabrizio Antonio Viola, Felix J. Berger, Nicolas F. Zorn, Jana Zaumseil, and Mario Caironi. "A rapidly stabilizing water-gated field-effect transistor based on printed single-walled carbon nanotubes for biosensing applications." *ACS Applied Electronic Materials* 3, no. 7 (2021): 3106-3113.

[4] Blusiewicz, K., G. Rydzewska, and A. Rydzewski. "Gastric juice ammonia and urea concentrations and their relation to gastric mucosa injury in patients maintained on chronic hemodialysis." *Rocz Akad Med Bialymst* 50 (2005): 188-192.

[5] Waring, A. J., I. M. Drake, C. J. Schorah, K. L. White, D. A. Lynch, A. T. Axon, and M. F. Dixon. "Ascorbic acid and total vitamin C concentrations in plasma, gastric juice, and gastrointestinal mucosa: effects of gastritis and oral supplementation." *Gut* 38, no. 2 (1996): 171-176.

[6] Beer, Kari A. Santoro, Rebecca S. Syring, and Kenneth J. Drobatz. "Evaluation of plasma lactate concentration and base excess at the time of hospital admission as predictors of gastric necrosis and outcome and correlation between those variables in dogs with gastric dilatation-volvulus: 78 cases (2004–2009)." *Journal of the American Veterinary Medical Association* 242, no. 1 (2013): 54-58.

[7] Annese, V.F., Patil, S.B., Hu, C., Christos Giagkoulovits, Al-Rawhani, M.A., Grant, J., Macleod, M., Clayton, D.J., Heaney, L.M., Daly, R., Accarino, C., Shah, Y.D., Cheah, B.C., Beeley, J., Jeffry, T.R., Jones, R., Barrett, M.P., and David (2021) A monolithic single-chip point-of-care platform for metabolomic prostate cancer detection. *Microsystems & Nanoengineering*, 7 (1).

[8] Barber, Robert, James Davis, and Pagona Papakonstantinou. "Stable chitosan and prussian blue-coated laser-induced graphene skin sensor for the electrochemical detection of hydrogen peroxide in sweat." *ACS Applied Nano Materials* 6, no. 12 (2023): 10290-10302.

[9] Walimbe, Pooja D., Rajeev Kumar, Amit Kumar Shringi, Obed Keelson, Hazel Achieng Ouma, and Fei Yan. "Electrochemical Detection of H2O2 Using Bi2O3/Bi2O2Se Nanocomposites." *Nanomaterials* 14, no. 19 (2024): 1592.

[10] Fukada, K., Tajima, T., and Seyama, M. (2022) Food-Based Capacitive Sensors Using a Dynamic Permittivity Change with Hydrogels Responsive to Hydrogen Peroxide. *Advanced Materials Technologies*, 7 (12).

[11] Zhuang, Z., Huang, L.L., Seo, B.C., Pandey, S., Karp, J.M., Lee, Y., and Maikawa, C.L. (2025) A radically simple, ingestible colorimetric biosensor pill for cost-effective, non-invasive monitoring of intestinal inflammation. *Device*, 100865–100865.

[12] Inda, M., Jimenez, M., Liu, Q., Phan, N., Ahn, J., Steiger, C., Wentworth, A., Riaz, A., Zirtiloglu, T., Wong, K., Ishida, K., Fabian, N., Jenkins, J., Kuosmanen, J., Madani, W., McNally, R., Lai, Y., Hayward, A., Mimee, M., and Nadeau, P. (2022) Ingestible capsule for detecting labile inflammatory biomarkers in situ.

[13] Kadian, S., Gopalakrishnan, S., Selvamani, V., Khan, S., Meyer, T., Thomas, R., Rana, M.M., Irazoqui, P.P., Verma, M.S., and Rahimi, R. (2024) Smart Capsule for Targeted

Detection of Inflammation Levels Inside the GI Tract. *IEEE Transactions on Biomedical Engineering*, 71 (5), 1565–1576.

[14] 5. Rajendran, S.T., Huszno, K., Dębowski, G., Sotres, J., Ruzgas, T., Boisen, A., and Zór, K. (2021) Tissue-based biosensor for monitoring the antioxidant effect of orally administered drugs in the intestine. Bioelectrochemistry, 138, 107720.

[15] Li, Yanzhen, Xin Lai, Yonggui Song, Ming Yang, Zhifu Ai, Yali Liu, Zhaozhi Qiu, Zexie Li, Huihui Liang, and Genhua Zhu. "Electrochemical, Real-Time Monitoring of Hydrogen Peroxide in Drosophila (Fruit Fly) Intestines Using a Carbon Fiber Microelectrode (CFME) Modified with Platinum Nanoparticles (PtNPs)." Analytical Letters 58, no. 7 (2025): 1214-1224.
